# Immunogenicity Testing of Lipidoids *In Vitro* and *In Silico*: Modulating Lipidoid-Mediated TLR4 Activation by Nanoparticle Design

**DOI:** 10.1016/j.omtn.2018.02.003

**Published:** 2018-02-13

**Authors:** Anne Marit de Groot, Kaushik Thanki, Monique Gangloff, Emily Falkenberg, Xianghui Zeng, Djai C.J. van Bijnen, Willem van Eden, Henrik Franzyk, Hanne M. Nielsen, Femke Broere, Nick J. Gay, Camilla Foged, Alice J.A.M. Sijts

**Affiliations:** 1 Department of Infectious Diseases and Immunology, Utrecht University, Utrecht, the Netherlands; 2 Department of Pharmacy, Faculty of Health and Medical Sciences, University of Copenhagen, 2100 Copenhagen, Denmark; 3 Department of Biochemistry, University of Cambridge, Cambridge, UK; 4 Department of Drug Design and Pharmacology, Faculty of Health and Medical Sciences, University of Copenhagen, 2100 Copenhagen, Denmark

**Keywords:** lipidoids, lipid-polymer hybrid nanoparticles, TLR4, immune modulation, stable nucleic acid lipid particles, SNALPs, lipoplexes, molecular modeling

## Abstract

Therapeutics based on small interfering RNA (siRNA) have promising potential as antiviral and anti-inflammatory agents. To deliver siRNA across cell membranes to reach the RNAi pathway in the cytosol of target cells, non-viral nanoparticulate delivery approaches are explored. Recently, we showed that encapsulation of siRNA in lipid-polymer hybrid nanoparticles (LPNs), based on poly(DL-lactic-co-glycolic acid) (PLGA) and cationic lipid-like materials (lipidoids), remarkably enhances intracellular delivery of siRNA as compared to siRNA delivery with LPNs modified with dioleoyltrimethylammoniumpropane (DOTAP) as the lipid component. However, the potential immune modulation by these cationic lipids remains unexplored. By testing lipidoids and DOTAP for innate immune-receptor-activating properties *in vitro*, we found that neither lipidoids nor DOTAP activate human Toll-like receptor (TLR) 2, 3, 7, and 9. However, in contrast to DOTAP, lipidoids are strong agonists for TLR4 and activate murine antigen-presenting cells *in vitro*. This agonistic effect was further confirmed *in silico* using a prediction model based on crystal structures. Also, lipidoids formulated as lipoplexes or as stable nucleic acid lipid particles, which was the reference formulation for siRNA delivery, proved to activate TLR4. However, by combining lipidoids with PLGA into LPNs, TLR4 activation was abrogated. Thus, lipidoid-mediated TLR4 activation during siRNA delivery may be modulated via optimization of the formulation design.

## Graphical Abstract

**Figure:**
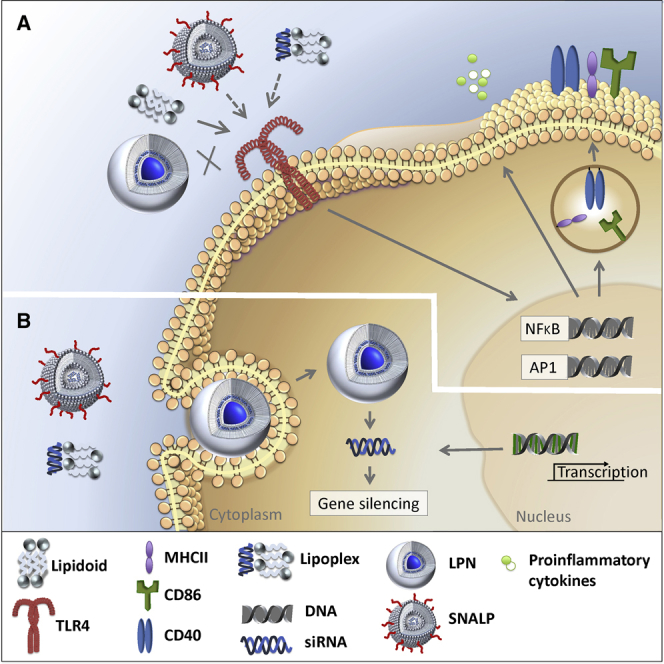

## Introduction

Small interfering RNA (siRNA) holds a highly promising therapeutic potential for the treatment of a variety of diseases via gene silencing.^1^, ^2^, ^3^ However, the physicochemical properties of siRNA often limit its therapeutic efficacy, rendering it unfavorable for efficient intracellular delivery to the RNAi pathway mediating gene silencing in the cytosol of the target cells. These properties include a relatively large molecular weight and high hydrophilicity as a result of the polyanionic phosphate backbone, leading to negligible cellular membrane permeation. Therefore, realization of the therapeutic potential of siRNA is fully dependent on the design and development of efficacious and safe siRNA delivery technologies.

Intracellular delivery of siRNA can be enhanced by employing nanoparticle-based delivery approaches.^4^, ^5^, ^6^, ^7^ Lipid-based delivery systems, in particular cationic lipids, have been shown to facilitate overcoming the critical obstacles to efficient siRNA delivery, which include pharmacokinetic barriers (a short circulation half-life due to susceptibility for nuclease degradation) and barriers at the cellular level (cellular uptake, intracellular trafficking, and endosomal escape to reach the cytosol).^5^ However, major drawbacks of commonly applied cationic lipids for siRNA delivery, e.g., 1,2-dioleoyl-3-trimethylammonium-propane (DOTAP), 1,2-di-O-octadecenyl-3-trimethylammonium propane (DOTMA), and dimethyldioctadecyl-ammonium bromide (DDAB), include (1) limited electrostatic interaction with siRNA as a result of the single quaternary ammonium head group (as opposed to structures containing multiple amine functionalities; see below), (2) activation of the innate immune system leading to undesired side effects, and (3) unfavorable biodistribution because of nonspecific tissue distribution and protein binding, eventually resulting in a relatively narrow therapeutic window.^8^, ^9^

Recent advances in the field of RNAi therapeutics have led to the identification of a novel class of synthetic cationic lipids, the so-called lipidoids, which are capable of mediating highly efficient intracellular delivery of siRNA.^10^, ^11^, ^12^, ^13^, ^14^ Lipidoids are lipid-like structures containing multiple secondary and tertiary amine functionalities, which confer highly efficient interaction with anionic siRNA molecules.^10^ Lipidoids have been formulated as long-circulating stable nucleic acid lipid particles (SNALPs), also containing cholesterol and PEGylated phospholipids, for intravenous administration.^10^, ^15^ The therapeutic efficacy of lipidoid-based SNALPs has been demonstrated in a number of *in vivo* models, but data from these studies also indicate potential symptoms of splenomegaly at higher doses.^10^ In addition, lipidoids, even in combination with control siRNA, can induce immunostimulatory effects.^16^ Several clinical trials involving SNALPs have been terminated owing to the occurrence of an influenza-like syndrome in the dosed patients.^17^, ^18^, ^19^ Premedication with corticosteroids is often necessary to circumvent such infusion-related problems.^20^, ^21^ Hence, there is an urgent need for the design and development of novel and safe drug delivery systems that circumvent the hurdles involved in intracellular delivery of siRNA. Recently, we recognized the potential of one specific type of nanocarrier, i.e., lipidoid-polymer hybrid nanoparticles (LPNs), for which highly promising results were demonstrated with respect to safe and efficient intracellular delivery of siRNA *in vitro*. These siRNA-loaded LPNs are composed of lipidoids and poly(DL-lactic-co-glycolic acid) (PLGA), and we recently reported highly systematic optimization of their critical quality attributes using a quality-by-design approach.^22^

Whereas gene silencing efficiency and effect on cell viability are often reported in the literature, the immune modulatory effects of siRNA and delivery systems remain poorly described. Potential immune modulatory effects are essential to investigate and identify already in the formulation development phase, and *in vitro* immunogenicity studies constitute an important technology platform in the early immunogenicity risk assessment of new formulations. Nucleic acids generally activate the innate immune system via binding to pattern-recognition receptors (PRRs), e.g., the Toll-like receptors (TLRs).^23^ Chemical modification of siRNA is commonly used to reduce undesired immune effects.^24^ However, functional excipients used for the siRNA delivery systems may also be recognized by the immune system because they adopt a structure similar to components of pathogens and hence stimulate a cascade of deleterious immune effects, which may lead to failure of therapy.^25^ So far, immunogenicity assessment of lipidoids has not been reported, albeit a thorough evaluation is warranted, considering their promising therapeutic potential.

The purpose of the present study was to assess the first-line *in vitro* immune modulatory effects of our previously synthesized and purified array of lipidoid compounds^22^ by using human TLR reporter cell-line-based assays and *in silico* modeling. These compounds have a tetra-amine backbone and comprise different degrees of alkylation: the tetra-acylated lipidoid (L_4_) consists of an acylated backbone displaying four acyl chains (isomeric mixture), the penta-acylated lipidoid (L_5_) carries an additional acyl chain (isomeric mixture), and the hexa-acylated lipidoid (L_6_) is fully acylated (Figure S1 depicts the most abundant isomers). We also tested a mixture of lipidoids with different degrees of alkylation, which constitutes the end product of the chemical synthesis reaction before purification,^22^ referred to as lipidoid mix (L_mix_). Furthermore, the immune modulatory effects of LPNs containing the different types of lipidoids (L_4_, L_5_, L_6_, and L_mix,_ respectively) were compared to those of lipoplexes, SNALPs, and DOTAP-modified LPNs.

## Results

### Bulk Lipidoids Activate TLR4 Responses

**Figure 1:**
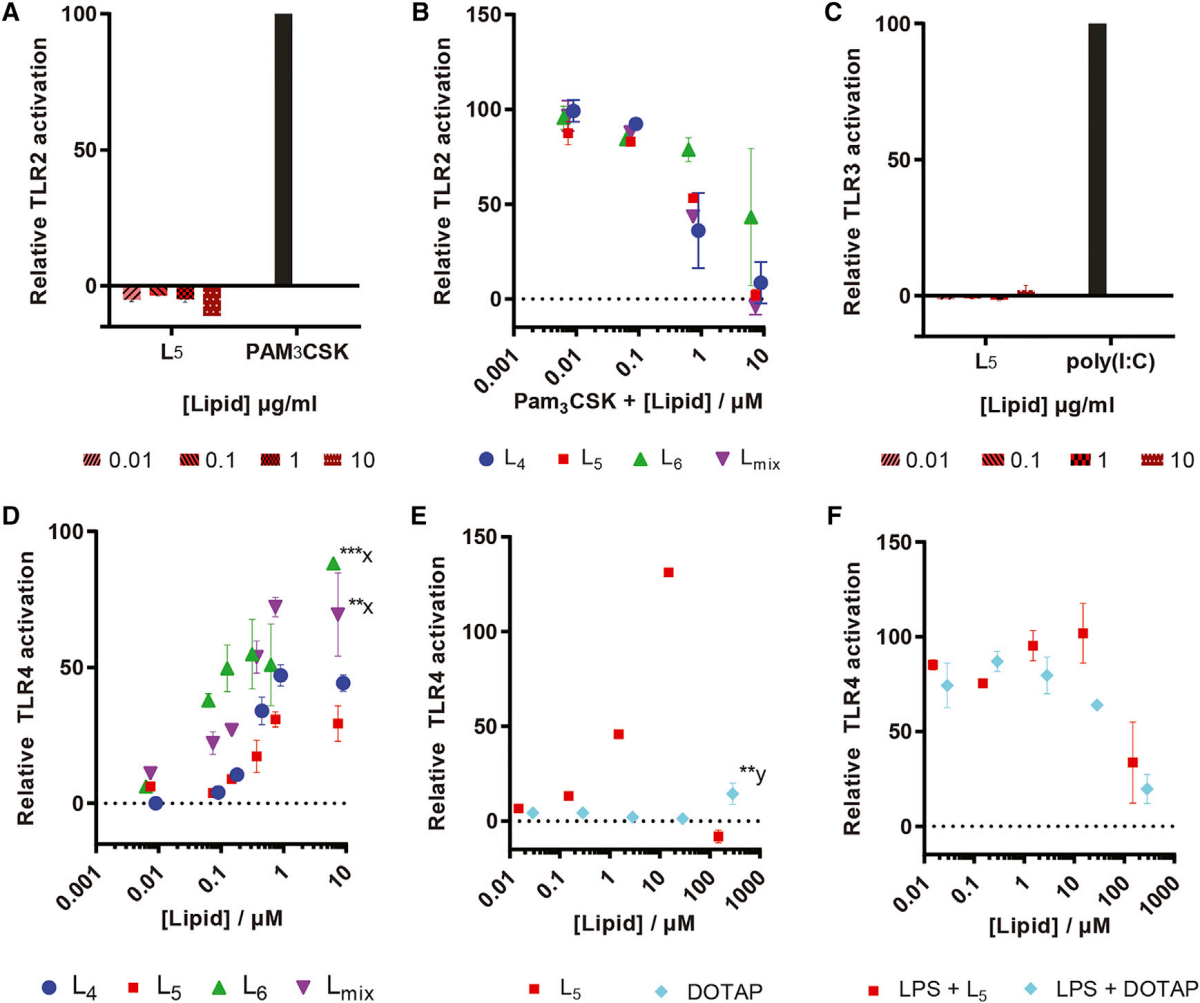
Lipidoid-Induced TLR Activation Assessed Using HEKblue Human TLR Reporter Cell Lines (A) Incubation of the TLR2-expressing cell line for 16 hr at 37°C with L_5_ and the positive control PAM_3_CSK_4_. (B) Incubation of the TLR2-expressing cell line with L_4_, L_5_, L_6_, and L_mix_ in the presence of PAM_3_CSK_4_, 100 ng/mL, is shown. (C) Incubation of the TLR3-expressing cell line with L_5_ and positive control poly(I:C) is shown. (D and E) TLR4-expressing cells were stimulated with L_4_, L_5_, L_6_, and L_mix_ concentrations ranging from 0.001 to 10 μM (D), as well as with L_5_ or DOTAP in the concentration range from 0.01 to 300 μM (E). (F) Activation of the TLR4-expressing cell line by L_5_ and DOTAP, respectively, in the presence of LPS, 10 ng/mL, is shown. Data have been corrected for background measured for solvent-stimulated cells and are shown relative to the maximal response (100%) with the respective selective agonists: PAM_3_CSK_4_ (100 ng/mL for TLR2; A and B), poly(I:C) (5 μg/mL for TLR3; C), and LPS (10 ng/mL for TLR4; D–F). Data represent mean values ± SD (n = 3; technical replicates) and represent results of one of two independent experiments. Statistically significant differences from L_5_ are marked with x and from solvent are marked with y: **p < 0.01 and ***p < 0.001.

The potential immunogenicity of lipidoids reported previously^10^ was evaluated by assessing the ability of the bulk compounds to activate the innate immune system via TLRs by using human TLR reporter cell lines. The lipidoids were tested for their ability to activate TLR2 using a reporter cell line expressing TLR2. No activation was noted upon incubation with L_5_, even at the highest tested concentration (10 μg/mL), whereas the positive control Pam_3_CSK_4_ activated TLR2 (Figure 1A). However, all lipidoids at concentrations above 1 μM did interfere with TLR2 activation, as demonstrated in the presence of the activator Pam_3_CSK_4_ (Figure 1B). The activity of L_5_ was further tested against TLR3, and no activation was noted (Figure 1C). The activity of L_5_ was further tested against TLR7 and TLR9, but no activation was measured either (data not shown).

In contrast, a concentration-dependent increase in the activation of TLR4 was noted for all lipidoids, i.e., L_4_, L_5_, L_6_, and L_mix_ up to 1 μM concentrations (Figure 1D). No significant difference in the activation of TLR4 was noted between L_4_ and L_5_ at the highest tested concentration (p value > 0.05). Overall, a statistically significant higher TLR4 activation was noted for L_6_ (p value < 0.001) and L_mix_ (p value < 0.01) as compared to L_5_, for which reason L_5_ was selected for further testing (Figures 1E and 1F). In contrast to L_4_–L_6_, DOTAP did not activate TLR4 in the tested concentration range up to 28.6 μM, although TLR4 activation was noted at the highest tested concentration of DOTAP (0.29 mM; p value < 0.01; Figure 1E). In order to test for potential assay interference, the experiments were also performed in the presence of the TLR4 agonist lipopolysaccharide (LPS). At higher concentrations, both L_5_ and DOTAP inhibited LPS-induced TLR4 activation (Figure 1F). This could be attributed to multiple factors, including loss of cell viability and/or lipidoid to ligand binding. Interference with the assay is a likely explanation for the reduced TLR4 activation observed at high L_5_ concentrations (above 1–10 μM lipidoid). Of note, at these high concentrations, the strongest TLR4 activation was noted for L_6_, which showed the least interference. Thus, the differences in TLR4 activating activity between L_4_, L_5_, and L_6_ at high concentrations could be explained by differential interference of the compounds with the assay. Thus, L_4_, L_5_, and L_6_ (at concentrations above 0.1 μM), but not DOTAP (up to 100 μM), activate TLR4, and interference with activation occurs at higher concentrations for all lipidoids tested.

### *In Silico* Prediction of Docking to TLR4/MD-2 Suggests that Lipidoids Are Potent Agonists

Lipidoids have been designed for optimal RNAi experiments. Their cationic head groups neutralize the anionic charges of the nucleic acids that they transport, whereas their hydrophobic moieties provide a scaffold for membrane fusion. These features also seem to induce interactions with the innate immune LPS sensor TLR4/MD-2. TLR4 is associated with myeloid differentiation factor 2 (MD-2) on the cell surface, and MD-2 is required for signaling.^26^ Whereas the number of acyl chains and the presence of phosphate groups are known key features for LPS recognition, we here explored the potential binding mode of the positively charged lipidoids containing tetra-amine backbones. We will refer to the head group amines as N1, N2, N3, and N4 in the following section. At pH 7.0, L_4_ is predicted to possess protonated head group amines in positions N1 and N3. Upon acidification, an additional protonation may take place on N4. The protonation state differs slightly in L_5_, wherein the presence of an extra acyl chain shifts the protonations to positions N2 and N4 at pH 7.0 and to N1, N2, and N4 under more acidic conditions (pH 6.0). In contrast, protonation is predicted to take place on the N1 and N4 amines at both pH 6.0 and pH 7.0, respectively, in case of L_6_. More acidic environments of pH 5.0 and below may trigger a third protonation at N2. The experimentally observed interaction of lipidoids with the TLR4 reporter cell line was further investigated by *in silico* docking studies. Docking was performed at pH 7.0 to compare binding affinities to MD-2 and TLR4 (Table 1) and to identify the binding modes for lipidoids (Tables S1, S2, and S3) using Autodock Vina software.^27^ DOTAP and other ligand complexes with known crystal structures were included as controls. These comprise of species-specific antagonist lipid Iva,^28^ the potent agonist lipid A of LPS from *Escherichia coli*,^29^ and the potent antagonist eritoran.^30^

**Table 1:** Predicted Binding Energies for Compounds to Human TLR4/MD-2 (at pH 7.0)

Ligand	Active hTLR4/MD-2 (3FXI)	Active hMD-2 (3FXI)	Inactive hMD-2 (2E59)
L_4_	−32.7	−31.1	−23.6
L_5_	−39.2	−36.5	−33.2
L_6_	−41.4	−38.3	−25.7
DOTAP	−22.6	−22.3	−11.1
Lipid IVa^a^	ND	−18.5^b^	−25.1
*E. coli* lipid A^a^	−34.7	−27.7	ND
Eritoran^a^	ND	−15.6^b^	−23.3

Values are given in kcal/mol. The binding energies to active hMD-2 are extrapolated from rigid docking of the ligand conformation observed in the crystal structures, 2E59 and 1Z65, respectively. ND, not determined.

a Binding energies are given for rigid docking to match the ligand conformations observed in the crystal structures.

b Lipid IVA and eritoran are antagonists.

The predicted binding energies suggest that lipidoids are notably more potent than other known TLR4 agonists, with a predicted binding energy of −32.7 kcal/mol for L_4_, −39.2 kcal/mol for L_5_, and −41.4 kcal/mol for L_6_, whereas a much lower binding energy was predicted for DOTAP (−22.6 kcal/mol; Table 1). The binding to MD-2 in inactive^28^ and active conformations,^29^ respectively, was also compared. For all crystal structures published to date, agonists seem to interact with the F126 loop of MD-2 that subsequently adopts a more compact conformation, enabling TLR4 dimerization.^29^ In contrast, in the absence of ligand or in the presence of an antagonist, this loop protrudes into the solvent, which prevents receptor dimerization, either directly by causing steric hindrance, or indirectly by not contributing to the dimerization interface.^30^ Hence, the predicted binding of L_5_ to active and inactive MD-2, respectively, was compared: the affinity was highest for the active MD-2 (−36.5 kcal/mol, as compared to −33.2 kcal/mol for inactive MD-2). The opposite was the case for lipid IVa, which is a known antagonist in humans: the affinity for the inactive conformation of MD-2 was −25.1 kcal/mol, as compared to −18.5 kcal/mol for the active MD-2. The affinity of DOTAP for inactive MD-2 was remarkably low (−11.1 kcal/mol), which suggests that the binding is probably not specific. Hence, data support that L_5_ is likely to bind and activate TLR4/MD-2, whereas DOTAP binding remains questionable. Interestingly, other tested lipidoids (L_4_ and L_6_) also exhibited similar profiles for binding energies to active and inactive TLR4/MD-2 (Table 1). Although, the difference in the predicted binding energies was relatively higher in case of L_4_ and L_6_, primary emphasis was given to L_5_ because of its relatively higher transfection potential.^22^

**Figure 2:**
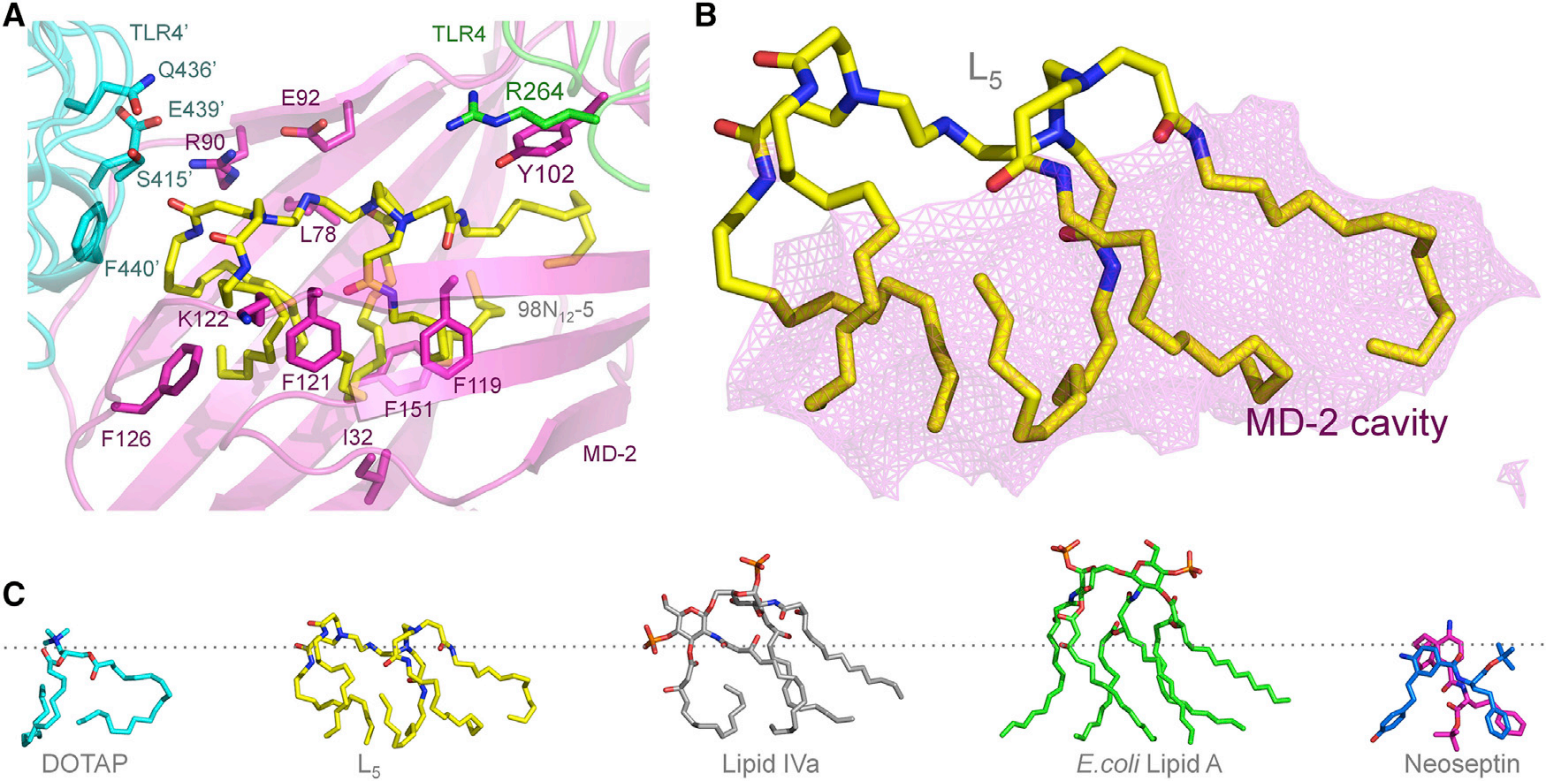
Binding Mode of L_5_ (A) Graphical representation of L_5_ (yellow) binding to TLR4/MD-2 with primary TLR4 (green), secondary TLR4 at the dimer interface denoted TLR4’ (cyan), and MD-2 (magenta). L_5_ and its closest protein contacts are shown as sticks with oxygen (red) and nitrogen (blue) atoms. (B) Close-up of L_5_ filling up the hydrophobic cavity of MD-2 is shown: the solvent-accessible pocket of MD-2 is represented as a semi-transparent pink mesh. (C) Different ligand conformations predicted for TLR4/MD-2 binding are shown: DOTAP, L_5_, and the species-specific antagonist lipid IVa as observed in complex with human MD-2 (PDB: 2E59); the potent agonist *E. coli* lipid A (PDB: 3FXI); and the species-specific agonist neoseptin, as observed in complex with mouse TLR4/MD-2 (PDB: 5HG4). The dotted line represents the same position of the ligand in the MD-2 cavity (see also Figure S3).

Further, although *E. coli* LPS is the most potent TLR4/MD-2 agonist, its lipid A moiety is predicted to bind with affinities comparable but lower than L_5_, which range from −27.7 kcal/mol for active MD-2 to −34.7 kcal/mol for the TLR4/MD-2 dimeric complex. This suggests that core sugars contribute to the activity of LPS. Tetra-acylated eritoran in contrast lacks sugars and resembles lipid IVa in terms of binding energies (−23.3 and −25.1 kcal/mol, respectively; Table 1). As for LPS, L_5_ binds in the hydrophobic cleft of MD-2, where it makes extensive contacts both with MD-2 and the TLR4 dimer (referred to as TLR4’; Figure 2A). The lack of direct interaction with TLR4 at the primary binding site is not uncommon and has also been described for neoseptin, which is another unconventional ligand.^31^ L_5_ is buried deeply in the MD-2 pocket (Figure 2B), where it is located much deeper than lipid IVa (Figure 2C), which is an antagonist in humans.^28^ Its positioning is partially overlapping the binding region described for a neoseptin dimer bound to mouse TLR4/MD-2.^31^ L_5_ is much larger than the neoseptin dimer and makes additional contacts throughout the hydrophobic cavity, involving in particular the MD-2 residues I32, L78, Y102, F119, F121, K122, F126, and F151, which surround the ligand and mediate the strongest van der Waals interactions within the complex. In contrast to neoseptin, which is only able to activate mouse TLR4/MD-2, cell-based assays show that L_5_ is active on both human and mouse proteins, probably as a result of the extensive hydrophobic network, which is conserved across species.

However, no strong hydrogen bonds or ionic interactions between L_5_ and TLR4/MD-2 could be predicted, implying a lack of specificity in binding. This is unusual for protein-ligand interactions and has to date only been described for DNA-ligand stacking interactions.^32^ The high flexibility of the L_5_ molecule may allow it to fit well into the hydrophobic binding pocket, mediating extensive hydrophobic contacts within the complex. Whereas hydrogen bond formation provides binding specificity and rigidity in molecular interactions, we cannot rule out that water molecules and counter ions may associate with the cationic headgroup.^33^ Potential hydrogen bonds and polar interactions are foreseen with neighboring residues from MD-2 (residues R90 and E92), TLR4 (R264), and at the dimer interface with TLR4’ at S415’, Q436’, and E439’ (Figure 2; Table S1). Whereas DOTAP binding to inactive MD-2 is nonspecific, binding to the active form of MD-2 nevertheless seems to be possible according to our docking predictions. However, DOTAP docks more than 10 Å from residue F126 and more than 15 Å from the dimerization interface when artificially associated to the active conformation of MD-2. Together with the lack of affinity of DOTAP for inactive MD-2, these data suggest that DOTAP does not possess any of the features essential for TLR4 activity, which is in sharp contrast to L_5_.

Docking studies further suggest that the hydrophobic moiety of L_4_ is sufficient for binding within the MD-2 cavity and for establishing stabilizing contacts with the F126 loop, which are required for receptor activation.^34^ Increasing the degree of acylation of the tetra-amine backbone (L_5_ and L_6_) promotes a more surface-exposed conformation of the lipidoids with additional hydrophobic and hydrophilic contacts (Table S2). In particular, L_6_ is the only tested lipidoid that is able to interact with TLR4 at the primary site as well as at the dimer interface (Table S3). Indeed, the R264 residue of TLR4 serves as a hydrogen bond donor to the carbonyl group of the acyl chain linked to N3. The additional contacts made by L_6_ contribute to its enhanced TLR4 activity, as compared to the less acylated lipidoids. All lipidoids studied here share conserved interactions with E439’ at the TLR4 dimer interface. The docking poses and binding affinities are in accordance with the observed TLR4 activity in the cell-based assays.

### Lipidoids Induce Maturation of Professional Antigen-Presenting Cells

**Figure 3:**
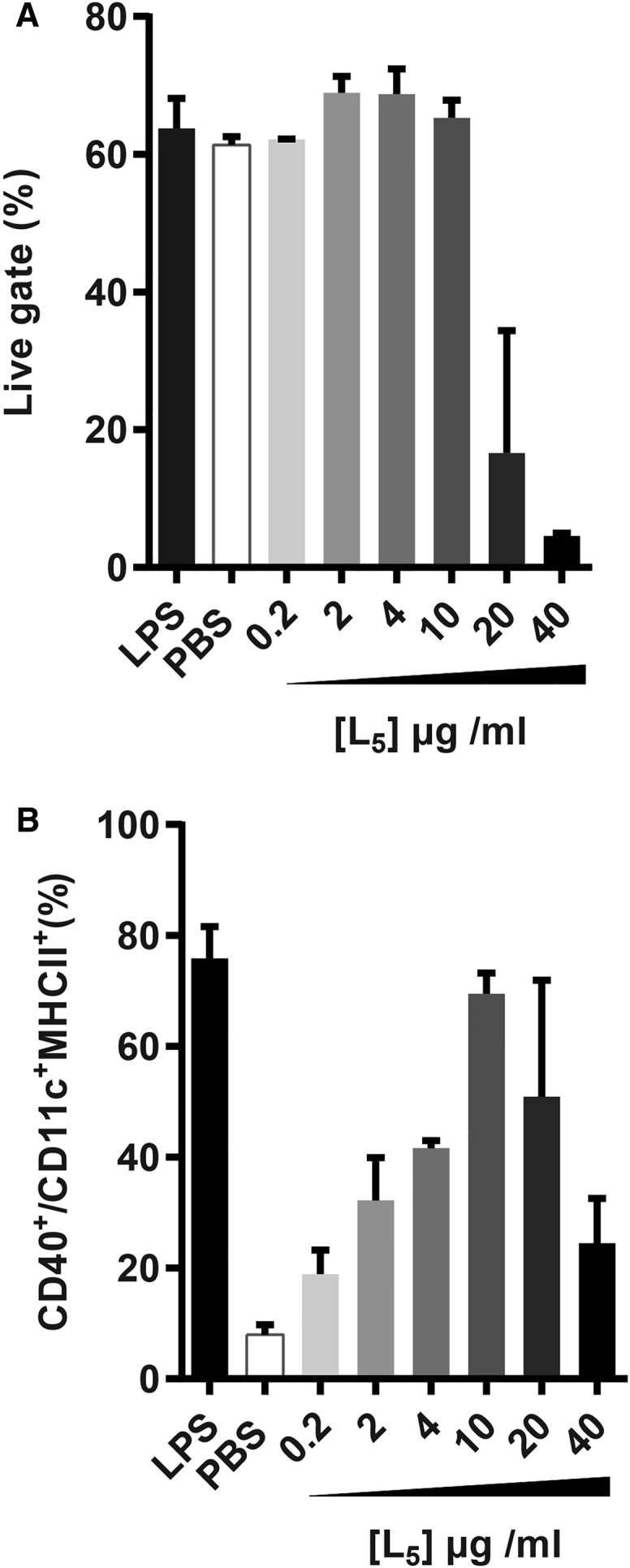
Lipidoid-Induced Cell Maturation Was Tested by Stimulating Murine Bone-Marrow-Derived pAPCs Differentiated with Granulocyte Macrophage Colony-Stimulating Factor (A) Cell viability is shown as the percentage of cells in the live gate quantified by flow cytometry upon stimulation with indicated concentrations of L_5_ and compared to the viability of BM-APCs incubated with LPS and PBS. (B) Maturation is shown, measured as the upregulation of the activation marker CD40 in CD11c^+^MHCII^+^ cells, and compared to CD40 levels on LPS (10 ng/mL) and PBS (1:25) incubated BM-APCs. Data represent mean values ± SE (n = 2; technical replicates) and represent results of one of a total of two independent experiments.

Besides induction of an inflammatory response, activation of the adaptive immune system by the designed siRNA delivery systems should also be avoided. Professional antigen-presenting cells (pAPCs) are initiators of adaptive responses, and upon encounter with drug delivery systems, they may prime cells of the adaptive immune system by inducing differentiation from an immature to a mature cell type.^35^ First, to test whether lipidoids affect viability and induce maturation of pAPCs, murine bone marrow (BM)-derived APCs were incubated with L_5_. A noticeable decrease in the viability of BM-APCs occurred upon treatment with L_5_ at concentrations above 10 μg/mL as compared to the viability of BM-APCs incubated with solvent or LPS alone (Figure 3A). Within the live-cell population, no difference was observed in the percentage of cells with a dendritic-like phenotype, measured as CD11c and major histocompatibility complex (MHC) class II double-positive cells. However, a concentration-dependent increase in maturation was noted in case of L_5_ up to 10 μg/mL, which was measured as an increase in the percentage of CD40^+^ cells (Figure 3B) and CD86^+^ cells (data not shown). Reduced maturation at higher concentrations could be attributed to cytotoxicity (Figure 3A). Thus, the tested lipidoids do not only activate the TLR4 pathway but do also induce maturation of BM-APCs.

### TLR4 Activation and pAPC Maturation Are Influenced by the Type of Formulation

**Figure 4:**
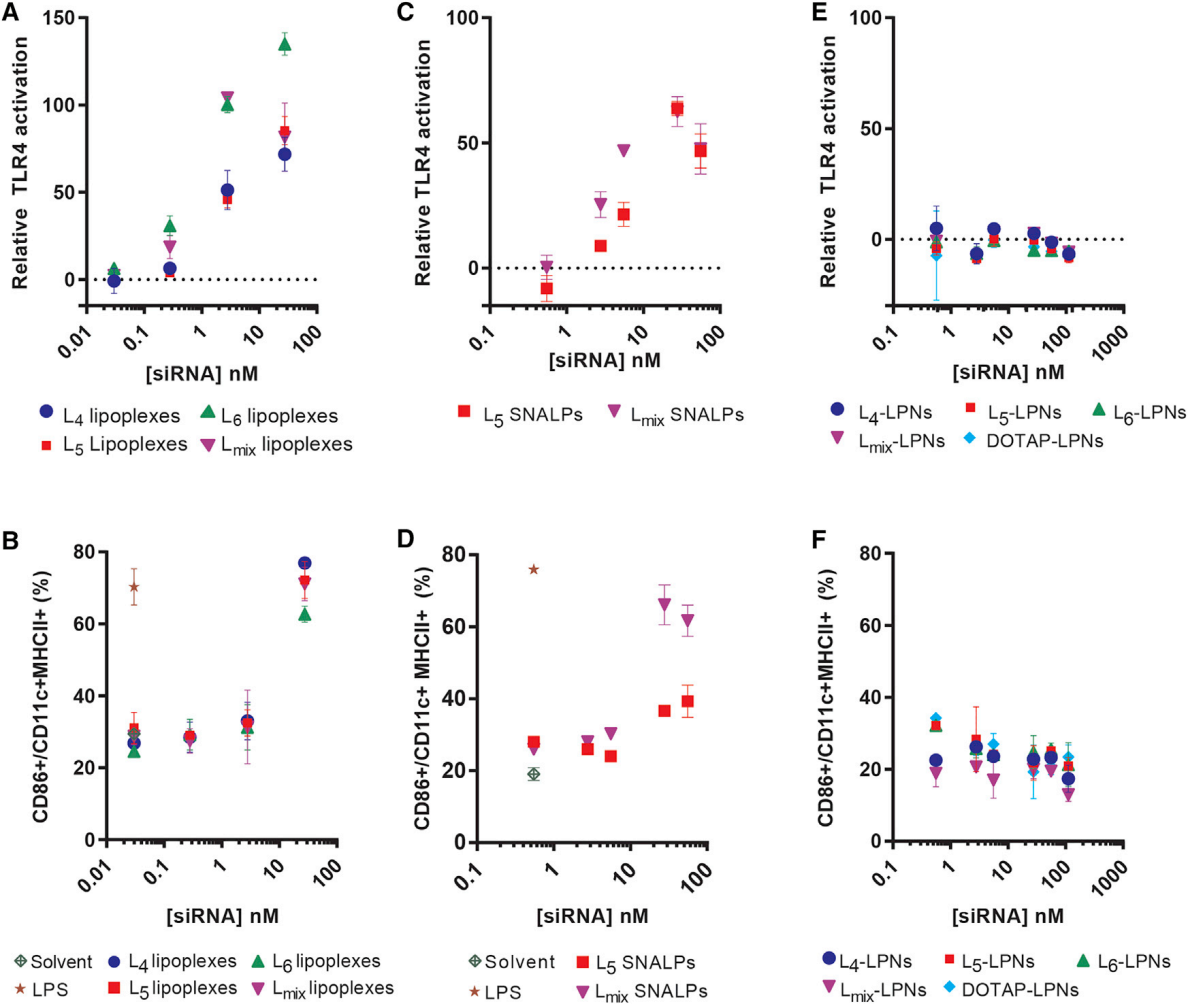
Immune Activation by Lipidoid-Based SNALPs and Lipoplex and Not by Lipidoid-Modified LPNs (A and B) Activation of the hTLR4 receptor cell line (A) and pAPC maturation (B) by lipoplexes composed of L_4_, L_5_, L_6,_ or L_mix_ with siRNA-EGFP. (C and D) Activation of the hTLR4 receptor cell line (C) and pAPC maturation (D) by SNALPs prepared with L_5_ and L_mix_, respectively, is shown. (E and F) Activation of the hTLR4 receptor cell line (E) and pAPC maturation (F) induced by LPNs prepared with L_4_, L_5_, L_6_, L_mix_, or DOTAP is shown. Sample OD was corrected for OD value measured for the negative control (solvent 1:10) and divided by the maximum OD (LPS 10 ng/mL). Professional APC maturation was tested by stimulating murine bone-marrow-derived, granulocyte macrophage colony-stimulating factor (GM-CSF)-differentiated pAPCs. Maturation was measured as the upregulation of the activation marker CD86 in CD11c^+^MHCII^+^ cells and compared to the levels on BM-APCs stimulated with LPS (10 ng/mL) and PBS, as quantified by flow cytometry (see also Figure S2). Data represent mean values ± SD (n = 3 for A, C, and E and n = 2 for B, D, and F technical replicates).

The influence of formulation type on the immune-modulating properties of lipidoids was subsequently assessed systematically by measuring TLR4 activation upon incubation with three different types of formulations of lipidoids, viz. lipoplexes, SNALPs, and LPNs. Lipoplexes represent the simplest type of formulation, and they were prepared by simple co-incubation of siRNA and lipidoids at a fixed ratio of 1:20 (w/w). A concentration-dependent increase in the relative TLR4 activation was measured for all types of lipoplexes, composed of L_4_, L_5_, and L_6_, respectively (Figure 4A). In case of lipoplexes containing L_mix_, a decrease in TLR4 activation was noted at the highest concentration of 27 nM, as compared to that measured at 2.7 nM, which could be attributed to the interference with the measurement of TLR4 activation associated with toxicity (data not shown). Furthermore, the pAPC maturation assay revealed that all tested lipoplexes were well tolerated till 2.7 nM, albeit significant maturation was noted in all cases at the highest concentration of 27 nM (Figure 4B). Interestingly, remarkably higher pAPC maturation was observed after incubation with bulk lipidoids (Figure 3B) compared to incubation with lipoplexes, suggesting an influence of siRNA complexation on the immune-stimulating properties of lipidoids.

In addition, SNALPs formulated with L_5_ and L_mix_, respectively, were prepared as described previously (Table S4).^10^ Both L_5_ SNALPs and L_mix_ SNALPs induced concentration-dependent TLR4 activation from 2.5 nM till 27 nM, beyond which a decrease in TLR4 activation was noted for both formulations (Figure 4C). This decrease can be attributed to the interference with the measurement of TLR4 activation (data not shown). Like lipoplexes, both types of SNALPs induced significantly higher pAPC maturation at concentrations above 2.7 nM, measured as the upregulation of the maturation marker CD86 on BM-APCs (Figure 4D).

Finally, a series of siRNA-loaded LPNs was prepared with lipidoids (L_4_, L_5_, and L_6_) and DOTAP as previously described (Table S4).^22^ No TLR4 activation could be measured for LPNs modified with the lipidoids up to 100 nM siRNA (Figure 4E), independently of the specific type of lipidoid used. Furthermore, LPNs modified with either lipidoid or DOTAP did not induce any upregulation of the maturation markers CD86 (Figure 4F), CD40, and MHCII (data not shown), indicating that they do not induce pAPC maturation (Figure 4F). Interestingly, the LPNs did not influence the viability of BM-APCs (Figure S2). Thus, as LPNs do not have any measurable immune-activating properties in the present studies, the LPN-based formulation approach may be a promising delivery technology for further development of siRNA-based therapies.

## Discussion

In previous studies we showed that both DOTAP-modified^36^ and in particular lipidoid-modified LPNs are promising delivery systems for siRNA, with an effective silencing of gene expression in cellular systems.^22^ However, to date very few studies have addressed the potential undesired immune activation mediated by siRNA delivery systems. In the present study, the immune-modulatory effects of newer generation cationic lipids, i.e., lipidoids and various formulations composed of lipidoids, were tested. The results clearly demonstrate that bulk lipidoids are able to specifically activate TLR4, inducing a pro-inflammatory environment, in contrast to the commonly used cationic lipid DOTAP. However, formulation as lipidoid-modified LPN substantially diminishes the lipidoid-mediated TLR4 activation.

In agreement with the measured TLR4 activation by the lipidoids *in vitro*, we found that L_4_, L_5_, and L_6_ fitted into the MD-2 groove when analyzing possible binding to TLR4/MD-2 *in silico*. Whereas L_5_ may be the most potent compound for siRNA transfection, L_6_ appeared to be the most immune-reactive structure. This structure dependency was expected because the most potent TLR4 activators known to date are bacterial hexa-acylated LPS molecules. Penta-acylated lipids, e.g., LPS from *Rhodobacter sphaeroides*,^37^ and tetra-acylated ones, for example, lipid IVa and eritoran, are either species-specific partial agonists or antagonists.^38^ Thus, lipidoids, including L_4_–L_6_, may adopt a conformation suitable for binding to MD2, which is a prerequisite for dimerization and activation of TLR4.^28^, ^29^ In contrast, DOTAP was not predicted to bind with high specificity to MD-2 and does therefore not qualify either as a TLR4 agonist or as an antagonist, based on the *in silico* modeling. Taken together, the *in silico* predictions correlate well with the measured TLR4-activating abilities of the different test substances. Thus, *in silico* modeling of structures seems to be useful to investigate the potential of lipid structures for TLR4 binding during early immunogenicity risk assessment.

Our comparison of the different types of L_5_-containing delivery systems (lipoplexes, SNALPs, and LPNs) with respect to TLR4 activation *in vitro* shows that lipoplexes and SNALPs activate TLR4, whereas formulation of L_5_ in LPNs reduces or even prevents lipidoid-mediated TLR4 activation. Thus, the *in vitro* data suggest that LPNs may provide a non-immunogenic modality for intracellular siRNA delivery at optimal dosing (see discussion below). L_5_-containing SNALPs are currently being developed for antiviral therapy.^15^, ^39^, ^40^ This differential activation of TLR4 may be explained by important differences in the physicochemical characteristics of the delivery systems. These include the way the lipidoid molecules are exposed on the surface of the nanoparticles and how the lipidoid-containing structures are presented to the receptor.^41^ Because SNALPs and LPNs are composed of different materials, the structure of the particles differs for the two particle types and, most likely, therefore also the way L_5_ is exposed on the particle surface. In addition, we hypothesize that LPS-binding protein (LBP), which is capable of extracting LPS from bacterial membranes,^42^, ^43^ may be able to access and bind lipidoid molecules present in the looser packed membrane bilayers of SNALPs, but not the lipidoid molecules of the more tightly packed shell membrane structure that we suggest coats the PLGA core of LPNs.^44^ As a consequence, the L_5_ component of the particles may interact differently with the MD-2/TLR4 molecules, depending on the specific particle structure. Further studies are needed to test this hypothesis.^45^

Although lipidoid-induced TLR4 activation represents a potential safety concern, formulating lipidoids into LPN structures appears to modulate lipidoid-mediated TLR4 activation in a beneficial direction. This suggests that lipidoid-containing LPNs may be a promising platform technology for siRNA delivery. Of particular importance is that a concentration of 2.6 nM siRNA delivered with L_5_-modified LPNs induced 50% gene silencing (inhibitory concentration 50 [IC_50_]) after 24 hr:^22^ at this time point (16–24 hr), we could not measure any detectable immune activation by the LPNs at siRNA concentrations as high as 100 nM. This indicates that cellular events (cellular uptake, intracellular trafficking, and endosomal escape) taking place during this time interval do apparently not result in exposure of L_5_ to TLR4. In contrast, SNALPs and lipoplexes activate TLR4 already at concentrations corresponding to 2.6 nM siRNA. These types of delivery systems mediate less efficient gene silencing than LPNs (IC_50_ values of 5.6 and 25.5 nM, respectively).^22^ Our previously reported *in vitro* gene silencing data and the present *in vitro* immunogenicity data thus suggest that the LPNs are the most promising of the tested siRNA delivery technologies. However, further studies are needed to investigate the effect of LPNs on immunogenicity at higher doses and longer exposure times. In addition, preclinical *in vivo* safety and efficacy studies are needed to investigate whether there is a correlation between these early immunogenicity risk assessment findings *in silico* and *in vitro* and the *in vivo* situation.

A few studies reported in the literature have investigated potential immune-activating effects of cationic lipid nanoparticles. Of particular interest is that, whereas bulk DOTAP and DOTAP-containing LPNs did not induce any immune activation in the present *in vitro* studies, DOTAP-containing liposomes did stimulate release of pro-inflammatory cytokines after *in vivo* administration in mice.^46^ This apparent difference may be attributed to the composition of the lipid-based nanoparticles: in contrast to the LPNs examined in the present study, the DOTAP-containing nanoparticles tested in the referred *in vivo* studies did also contain cholesterol and phosphatidylcholine, which may contribute to induction of an inflammatory response.^47^ In addition, species-specific differences in TLR4 activation (mouse versus human) may also influence the results.

We envisage that the differences in innate immune activation measured in the present study may be more actively exploited in the field of advanced drug delivery systems for nucleic-acid-based drugs. If the aim is to deliver an antiviral or an anticancer drug, specific immune activation may be a desirable outcome. The immune-stimulating effects of unmodified RNA in combination with SNALPs have already been described in the literature, and these effects likely contribute to efficacious antiviral therapy;^38^, ^39^ TLR4 activation mediated by the lipidoid component of SNALPs may contribute to that. In conclusion, the different possibilities we have with respect to design of drug delivery systems may provide a range of opportunities for customizing the formulations toward specific, desirable outcomes.

## Materials and Methods

### Materials

2′-O-methyl-modified dicer substrate asymmetric siRNA duplexes directed against EGFP (EGFP-siRNA) and scrambled negative control siRNA were synthesized by GlaxoSmithKline (Stevenage, UK) and provided generously as dried, purified, and desalted duplexes (Table S5). The siRNA stock solutions were prepared in 4-(2-hydroxyethyl)-1-piperazineethanesulfonic acid (HEPES) buffer (5 mM; pH 7.4) and re-annealed employing a standard protocol recommended by IDT (Coralville, IA, USA). PLGA with a lactide-to-glycolide molar ratio of 75:25 and a molecular weight of 20 kDa was procured from Wako Pure Chemical Industries (Osaka, JP). Polyvinyl alcohol (PVA) 403 with an 80.0% degree of hydrolysis was purchased from Kuraray (Osaka, JP). Cholesterol, DOTAP, and N-palmitoyl-sphingosine-1-succinyl[methoxy(polyethylene glycol)2000] (C_16_ PEG_2000_ ceramide) were obtained from Avanti Polar Lipids (Alabaster, AL, USA). Heparin, octyl-β-d-glucopyranoside (OG), and Tris–EDTA buffer (10 mM Tris and 1 mM EDTA [pH 7.5], referred to as TE buffer) were purchased from Sigma-Aldrich (St. Louis, MO, USA). RNase-free diethyl pyrocarbonate (DEPC)-treated Milli-Q water was used for all buffers and dilutions. All other chemicals, unless otherwise stated, were of analytical grade and obtained from local suppliers.

### Preparation of Lipoplexes

The array of lipidoids was synthesized, purified, and characterized as previously described.^22^ Lipoplexes were prepared by co-incubation of the lipidoids (L_4_, L_5_, L_6_, and L_mix_) and EGFP-siRNA. A stock solution (40 mg/mL) of the respective lipidoid was prepared by dissolving it in DMSO containing 1% (v/v) trifluoroacetic acid. Lipoplexes were prepared by addition of the stock solution to a siRNA solution in TE buffer at a lipidoid:siRNA ratio of 1:20 (w/w). The lipoplexes were vigorously vortexed before use. For L_mix_, the molecular weight of L_5_ was assumed for molarity calculations.

### Preparation of SNALPs

Lipidoid-based SNALPs were prepared as reference formulation for comparative purpose. A previously reported ethanol destabilization method^10^ was adopted^22^ and employed for preparation of L_5_-modified SNALPs and L_mix_-modified SNALPs. Briefly, an *in situ* buffer formation technique was used to formulate lipidoid-based SNALPs, considering its low solubility in EtOH. The lipidoid, cholesterol, and C_16_ PEG_2000_ ceramide were dissolved in EtOH at molar ratio of 42:48:10 using glacial acetic acid, and the mixture was added to an aqueous solution of NaOAc, resulting into formation of non-loaded SNALPs. The SNALPs were prepared at a lipid concentration of 10 mg/mL and loaded with EGFP-siRNA at a siRNA:lipid loading ratio of 1:7.5 (w/w) at 37°C for 30 min. The siRNA-loaded SNALPs were dialyzed against 1,000× volume PBS (pH 7.4) using a 100 kDa molecular weight cutoff dialysis membrane cassette (Float-A-Lyzer, G2; Spectrum Laboratories, Rancho Dominguez, CA, US) for 2 hr at room temperature to remove excess EtOH and un-entrapped siRNA. The detailed protocol for the preparation of SNALPs has been reported elsewhere.^22^

### Preparation of LPNs

Lipidoid- and DOTAP-modified LPNs were prepared by using the double emulsion solvent evaporation method, as reported previously,^48^ but with a slight modification.^22^ Briefly, an aqueous phase (w_1_) comprising the required amount of EGFP-siRNA in 125 μL HEPES buffer (5 mM; pH 7.4) was added to an organic phase (o; 250 μL CH_2_Cl_2_) containing PLGA and lipidoid or DOTAP. The formed primary emulsion was subjected to probe sonication at an amplitude of 50 (Misonix; Qsonica, CT, USA) in an ice bath. The primary w_1_/o emulsion was phase inversed by addition of 1 mL of 2% (w/v) PVA solution and vigorous vortexing for 1 min. The formed secondary w_1_/o/w_2_ double emulsion was subsequently subjected to probe sonication at an amplitude of 50 for additional 60 s. The size-reduced emulsion was transferred to a 25-mL beaker, and 5 mL of 2% (w/v) PVA solution was added under stirring. The stirring was continued for 45 min to facilitate the evaporation of the organic solvent. The prepared LPNs were then washed, purified, and lyophilized as reported previously.^22^ The lipidoid-modified LPNs were prepared at a lipidoid content of 15% (w/w) of the total solid content, whereas DOTAP-modified LPNs were prepared at 10% (w/w). Furthermore, the siRNA:lipidoid weight ratio was kept constant at 1:20 in case of lipidoid-modified LPNs, whereas it was 1:10 (w/w) for the DOTAP-modified LPNs.

### Physicochemical Characterization

The formulations were characterized with respect to hydrodynamic diameter, polydispersity index (PDI), zeta potential, and siRNA entrapment efficiency as previously described.^22^

In brief, a dynamic light scattering technique employing photon correlation spectroscopy (Zetasizer Nano ZS; Malvern Instruments, Worcestershire, UK) was used to measure the particle size and PDI of the formulations. Furthermore, the same samples were also used to measure the zeta potential employing the principles of laser Doppler micro-electrophoresis. The data for three independent batches were recorded and analyzed using the Zetasizer Software version 7.11 (Malvern Instruments).

The siRNA entrapment efficiency of LPNs and SNALPs was evaluated using a previously reported procedure.^22^, ^48^ Briefly, siRNA was extracted from the formulations by high-speed centrifugation and resuspension in CHCl_3_, and 100 μM OG and heparin were added to efficiently extract siRNA. Subsequently, after centrifugation, siRNA was quantified in the aqueous phase of the solution using the Quant-iT RiboGreen RNA Assay Kit (Molecular Probes, Invitrogen, Paisley, UK) according to the manufacturer’s protocol. The encapsulation efficiency and practical loading were calculated using the following equations:

(Equation 1) $$Encapsulation\;efficiency=\frac{Amount\;of\;entrapped\;siRNA}{Total\;amount\;of\;added\;siRNA}\times 100$$

(Equation 2) $$Practical\;loading=\frac{Amount\;of\;entrapped\;siRNA}{Total\;weight\;of\;nanoparticles}\times 100.$$

### Toll-like Receptor Activation Assays

The human TLR reporter cell lines (HEK-BluehTLR2, -hTLR3, -hTLR4, -hTLR7, and -hTLR9 reporter cells) were cultured according to the manufacturer's instructions (Invivogen, Toulouse, FR). Activation of hTLRs was measured according to the previously reported protocol.^49^ In brief, the reporter cell line assays were performed in serum-containing cell culture medium. Cells were seeded in flat 96-well plates and stimulated with different dilutions of fully dispersed lipids, lipoplexes, LPNs, and SNALPs, respectively, for 16 hr at 37°C, after which the cell culture supernatants were incubated with protein substrate QUANTIBlue (Invivogen) for 1 hr. Absorption was measured using a microplate reader (Bio-Rad 550; Bio-Rad, CA, USA) at optical density 650 (OD_650_) nm. The control value (solvent incubated) was subtracted from sample value. The relative alkaline phosphatase levels were then defined relative to the maximum TLR activation by the corresponding agonist.

### BM-APC Differentiation and APC Maturation Assays

Murine BM-APCs were differentiated with GM-CSF and cultured according to a reported protocol.^49^ Briefly, BM-APCs were stimulated with PBS (1:100 or 1:25; v/v), LPS (O127:B8; 10 ng/mL; Sigma-Aldrich, MO, USA), or with a concentration series of fully dispersed lipids, lipoplexes, LPNs, or SNALPs, respectively, for 16 hr at 37°C. Maturation was measured by antibody staining of the maturation markers CD40 and CD86, and the samples were analyzed using flow cytometry. Ethical approval for the mouse experiment was obtained from the Animal Experiment Committee of Utrecht University, the Netherlands, no. DEC.2013.II.09.102.

### Molecular Modeling Studies

The atomic coordinates of DOTAP and L_4_, L_5_, and L_6_ lipidoids were generated using the Sybyl software, a general molecular modeling program from Tripos, for which a partial license was donated to the Department of Biochemistry, University of Cambridge, UK.^50^ Briefly, Sybyl’s sketching tool was used to build the molecules, followed by a clean-up, which performs rough dynamics. Constraints were applied to bond lengths, angles, and torsions. The geometry of the molecules was optimized using the Powell minimization method with initial optimization based on the Simplex method, with a gradient of 0.05 kcal/mol and a maximum of 100 cycles of iteration. Partial charges were computed based on the Gasteiger-Hückel charge method using calculator plugin of Marvin Suite (version 17.2.20; ChemAxon, Budapest, Hungary).^51^ AutoDockTools4^52^ was used to convert the atomic coordinate files into a PDB partial charge, Q, and atom type, T, (PDBQT) format that contains the partial charges and atom types of the molecules, in addition to the atomic coordinates.^52^

For docking studies, the human crystal structures of lymphocyte antigen 96 (also known as MD-2) in the absence and presence of TLR4 (PDB: 2E59 and PDB: 3FXI, respectively) were used for docking upon removal of the ligands bound to the protein complexes. The potential ligands (DOTAP and L_5_) were docked into MD-2 using Autodock Vina.^27^ The protein component was kept rigid, whereas the ligand was allowed full flexibility. The Autogrid parameters were computed on an initial grid size of 32 × 32 × 32 Å^3^, with a spacing of 1 Å. The grid was centered on MD-2 at x = +22.626; y = −9.61; z = +15.271. The default optimization parameters were used for the iterated search in Vina, with a value of 32 for exhaustiveness. Flexible docking performed with DOTAP and lipidoid ligands generated a number of poses, ranked according to their binding energies. The highest energy poses predicting to the best binding mode according to the Vina algorithm were then subjected to rigid docking in order to compare the binding affinity of molecules of different sizes. The conformation of the docked ligands and their contacts with MD-2 and TLR4, where appropriate, were analyzed using PISA.^53^ Structural images were generated in PyMol (https://pymol.org/2) and Chimera.^54^ Detailed interactions were also analyzed using LigPlot+.^55^ Control docking experiments were performed on ligands with known crystal structures to compare their binding energies to lipidoids and DOTAP molecules. Controls included a potent TLR4/MD-2 agonist: the lipid A moiety of *Escherichia coli* LPS,^29^ a species-specific antagonist: lipid Iva,^28^ and a potent antagonist: eritoran.^30^ Rigid docking of these control ligands provided the binding energies that directly related to the experimental conformations adopted by these molecules.

### Statistics

Results are expressed as mean values ± SD. Statistical analysis was performed using GraphPad Prism (GraphPad, La Jolla, CA, USA). Statistically significant differences were assessed by ANOVA followed by a Dunnett’s multiple comparison test; p value below 0.05 was considered statistically significant. Significance of the results is indicated according to p values (*p < 0.05, **p < 0.01, and ***p < 0.001). A p value below 0.05 was considered statistically significant.

## Author Contributions

Conceptualization, A.M.d.G., K.T., C.F., and A.J.A.M.S.; Methodology, A.M.d.G., K.T., M.G., H.F., C.F., N.J.G., H.M.N., and A.J.A.M.S.; Investigation, A.M.d.G., K.T., D.C.J.v.B., M.G., E.F., and X.Z.; Writing – Original Draft, A.M.d.G., K.T., C.F., and M.G.; Writing – Review & Editing, A.M.d.G., K.T., C.F., A.J.A.M.S., W.v.E., M.G., H.M.N., and H.F.; Funding Acquisition, A.J.A.M.S., W.v.E., C.F., H.M.N., M.G., and N.J.G.; Supervision, A.J.A.M.S., F.B., N.J.G., H.F., H.M.N., and C.F.

## Conflicts of Interest

K.T., X.Z., H.F., H.M.N., and C.F. are co-inventors of a patent application relating to the cationic lipid structure (European patent application no. 16160931.8). All rights have been assigned to University of Copenhagen. All other authors report no potential conflicts.

## Supplemental Information

Document S1. Figures S1–S3 and Tables S1–S5

Document S2. Article plus Supplemental Information
