# Supplementary material for: Immunogenicity Testing of Lipidoids In Vitro and In Silico: Modulating Lipidoid-Mediated TLR4 Activation by Nanoparticle Design
Source: Mol Ther Nucleic Acids. 2018 Feb 13;11:159–69. doi: 10.1016/j.omtn.2018.02.003 (PMC5992342; doi:10.1016/j.omtn.2018.02.003)
Supplement: Document S1. Figures S1–S3 and Tables S1–S5 [file mmc1.pdf]

OMTN, Volume *11*

Supplemental Information

Immunogenicity Testing of Lipidoids *In Vitro* and *In Silico*: Modulating Lipidoid-Mediated TLR4 Activation by Nanoparticle Design

Anne Marit de Groot, Kaushik Thanki, Monique Gangloff, Emily Falkenberg, Xianghui Zeng, Djai C.J. van Bijnen, Willem van Eden, Henrik Franzyk, Hanne M. Nielsen, Femke Broere, Nick J. Gay, Camilla Foged, and Alice J.A.M. Sijts

Figure S1: **Chemical structures of L_4, L_5, L_6 and DOTAP**. L_4 and L_5 constitute a mixture of different isomers with identical molecular weight, and the most abundant L_4 and L_5 isomers are shown.

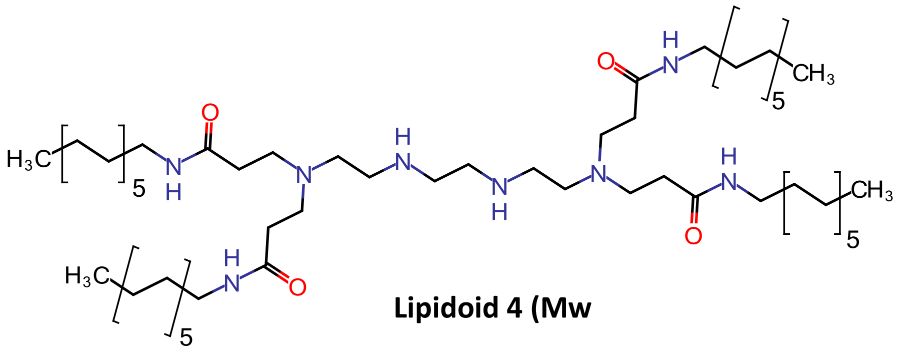

Lipidoid 4 (Mw

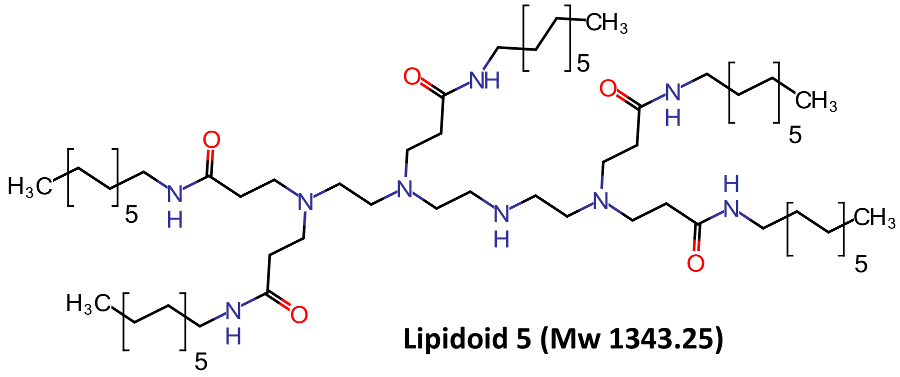

Lipidoid 5 (Mw 1343.25)

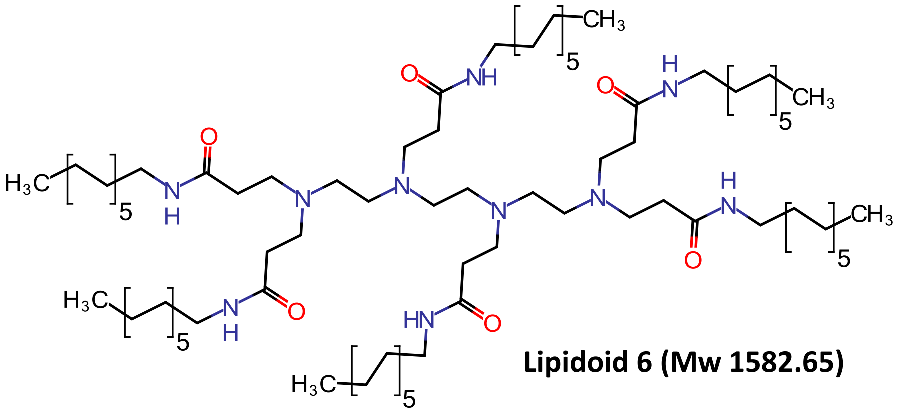

Lipidoid 6 (Mw 1582.65)

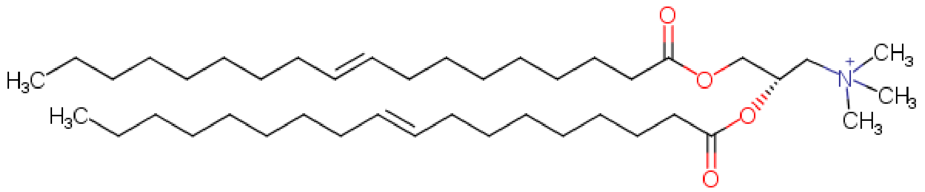

DOTAP (Mw 698.54)

Figure S2: **Lipidoid-polymer hybrid NPs have no effect on cell viability of BM-APCs.** Cell viability is shown as the percentage of cells in the live gate, quantified by flow cytometry upon stimulation with different concentrations of L_5, compared to BM-APCs incubated with LPS and PBS.

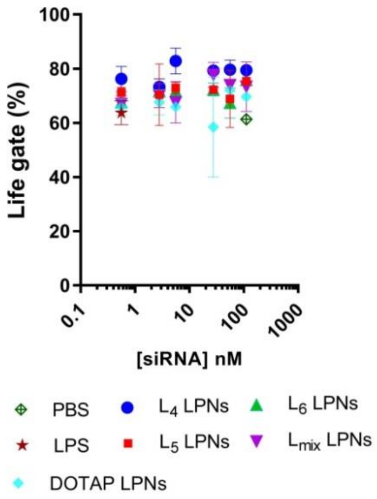

Figure S3: Proposed binding mode of lipidoids (L_4, L_5 and L_6), compared to lipid A moiety of LPS, to TLR4-MD-2. Top panels from left to right: L_4, L_6 and lipid A from LPS form hydrophilic contacts with TLR4 R264 at the primary binding site, MD-2 R90 and E92, which also form an ionic interaction with TLR4' E439' at the dimeric interface. TLR4 is represented in green cartoon, TLR4' in cyan and MD-2 in magenta. Bottom series from left to right: side views of L_4, L_5, L_6 and lipid A from LPS (TLR4 and MD-2 are not shown). The lipidoids of increasing acyl chain number spread their acyl chains to mimic lipid A conformation adopted in contact with the TLR4-MD-2 complex [29,28].

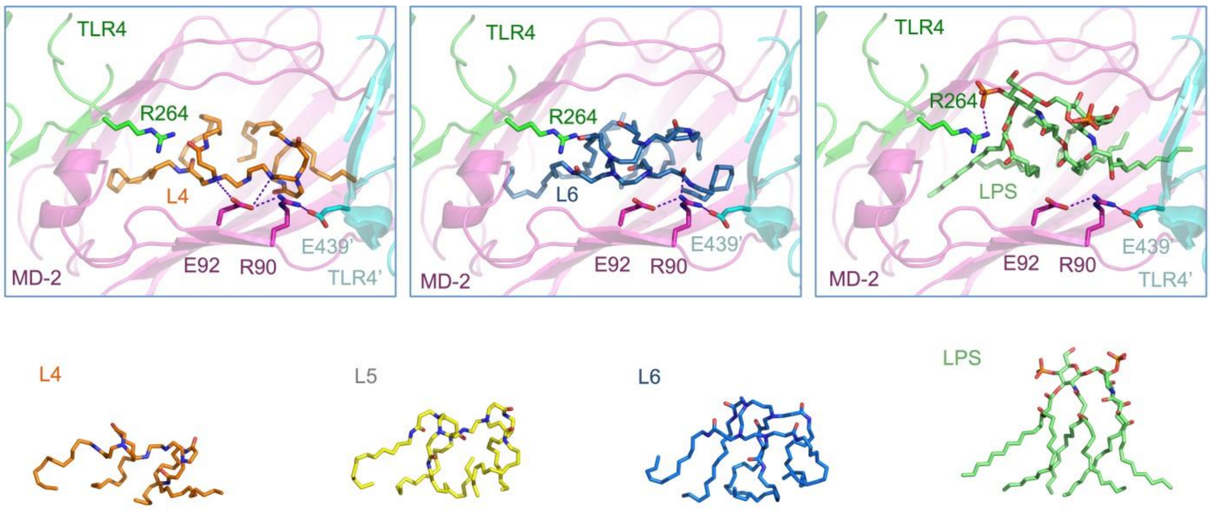

Table S1: TLR4-MD-2 contacts to L_4

List of residues from TLR4-MD-2 at given distances from L_4, according to the best vina docking model. MD-2 R90 is neutralized by E92 on one side and TLR4 is neutralized by E439' at the dimer interface (both are at 3.2 A). The same residues are in close proximity to the protonated amines of L_4 at positions 1 and 3. L_4 is more distant to the MD-2 residue F126, which undergoes a conformational change upon activation, as compared to L_5 and L_6. In contrast, L_4 is localized closer to the dimeric interface than L_5 and L_6. This proximity is illustrated below by the presence of TLR4 dimer residues (labelled TLR4').

**3.5 Å**	**4.0 Å**	**4.5 Å**	**5.0 Å**
	TLR4' E439	TLR4' E439	TLR4' E439
	TLR4' F440	TLR4' F440	TLR4' F440
	TLR4' S441	TLR4' S441	TLR4' S441
		TLR4' F463	TLR4' F463
MD-2 I32	MD-2 I32	MD-2 I32	MD-2 I32
MD-2 L61	MD-2 I46	MD-2 I44	MD-2 I44
MD-2 I63	MD-2 V48	MD-2 I46	MD-2 I46
MD-2 L78	MD-2 I52	MD-2 V48	MD-2 V48
MD-2 I80	MD-2 L54	MD-2 I52	MD-2 I52
MD-2 R90	MD-2 L61	MD-2 L54	MD-2 L54
MD-2 I94	MD-2 I63	MD-2 L61	MD-2 L61
MD-2 F104	MD-2 Y65	MD-2 I63	MD-2 I63
MD-2 F119	MD-2 L71	MD-2 Y65	MD-2 Y65
MD-2 I124	MD-2 F76	MD-2 L71	MD-2 L71
MD-2 Y131	MD-2 L78	MD-2 L74	MD-2 L74
MD-2 I153	MD-2 I80	MD-2 F76	MD-2 F76
	MD-2 V82	MD-2 L78	MD-2 L78
	MD-2 L87	MD-2 I80	MD-2 I80
	MD-2 R90	MD-2 V82	MD-2 V82
	MD-2 E92	MD-2 L87	MD-2 L87
	MD-2 I94	MD-2 R90	MD-2 R90
	MD-2 F104	MD-2 E92	MD-2 E92
	MD-2 I117	MD-2 I94	MD-2 I94
	MD-2 F119	MD-2 Y102	MD-2 Y102
	MD-2 F121	MD-2 F104	MD-2 F104
	MD-2 I124	MD-2 V113	MD-2 V113
	MD-2 F126	MD-2 T115	MD-2 T115
	MD-2 Y131	MD-2 I117	MD-2 I117
	MD-2 C133	MD-2 S118	MD-2 S118
	MD-2 F147	MD-2 F119	MD-2 F119
	MD-2 F151	MD-2 F121	MD-2 S120
	MD-2 I153	MD-2 I124	MD-2 F121
		MD-2 F126	MD-2 K122
		MD-2 Y131	MD-2 I124
		MD-2 C133	MD-2 F126
		MD-2 F147	MD-2 Y131
		MD-2 F151	MD-2 C133
		MD-2 I153	MD-2 L146
			MD-2 F147
			MD-2 F151
			MD-2 I153

Table S2: L_5 binding mode to TLR4-MD-2

List of residues from TLR4-MD-2 at given distances from L_5, according to the best vina docking model.

**3.5 Å**	**4.0 Å**	**4.5 Å**	**5.0 Å**
	TLR4’ E439	TLR4’ S415	TLR4’ S415
	TLR4’ F440	TLR4’ E439	TLR4’ E439
		TLR4’ F440	TLR4’ F440
MD-2 I32	MD-2 I32	MD-2 I32	MD-2 I32
MD-2 L78	MD-2 I44	MD-2 I44	MD-2 I44
MD-2 Y102	MD-2 I46	MD-2 I46	MD-2 I46
MD-2 F119	MD-2 V48	MD-2 V48	MD-2 V48
MD-2 F121	MD-2 I52	MD-2 I52	MD-2 I52
MD-2 K122	MD-2 L54	MD-2 L54	MD-2 L54
MD-2 F126	MD-2 L61	MD-2 L61	MD-2 L61
MD-2 F151	MD-2 I63	MD-2 I63	MD-2 I63
	MD-2 Y65	MD-2 Y65	MD-2 Y65
	MD-2 L71	MD-2 L71	MD-2 L71
	MD-2 L74	MD-2 L74	MD-2 L74
	MD-2 F76	MD-2 F76	MD-2 F76
	MD-2 L78	MD-2 L78	MD-2 L78
	MD-2 I80	MD-2 I80	MD-2 I80
	MD-2 R90	MD-2 L87	MD-2 L87
	MD-2 E92	MD-2 R90	MD-2 R90
	MD-2 Y102	MD-2 E92	MD-2 E92
	MD-2 F104	MD-2 I94	MD-2 I94
	MD-2 I117	MD-2 Y102	MD-2 Y102
	MD-2 F119	MD-2 F104	MD-2 F104
	MD-2 S120	MD-2 I117	MD-2 V113
	MD-2 F121	MD-2 S118	MD-2 I117
	MD-2 K122	MD-2 F119	MD-2 S118
	MD-2 I124	MD-2 S120	MD-2 F119
	MD-2 F126	MD-2 F121	MD-2 S120
	MD-2 Y131	MD-2 K122	MD-2 F121
	MD-2 C133	MD-2 G123	MD-2 K122
	MD-2 V135	MD-2 I124	MD-2 G123
	MD-2 F147	MD-2 F126	MD-2 I124
	MD-2 L149	MD-2 Y131	MD-2 F126
	MD-2 F151	MD-2 C133	MD-2 Y131
	MD-2 I153	MD-2 V135	MD-2 C133
		MD-2 L146	MD-2 V135
		MD-2 F147	MD-2 L146
		MD-2 L149	MD-2 F147
		MD-2 F151	MD-2 L149
		MD-2 I153	MD-2 F151
			MD-2 I153

Table S3: L_6 binding mode to TLR4-MD-2

TLR4 R264 is a hydrogen bond donor to the carbonyl group of the acyl chain linked to N3. L6 is the only lipidoid making a hydrophilic contact at the primary TLR4 site as well as at the dimer interface. Hydrophobic interactions are more extensive the bigger the lipidoid, but otherwise overlapping.

**3.5 Å**	**4.0 Å**	**4.5 Å**	**5.0 Å**
TLR4 R264	TLR4 R264	TLR4 R264	TLR4 R264
TLR4’ E439	TLR4’ E439	TLR4’ E439	TLR4’ Q436
TLR4’ F440	TLR4’ F440	TLR4’ F440	TLR4’ E439
			TLR4’ F440
			TLR4’ S441
MD-2 I32	MD-2 V24	MD-2 V24	MD-2 V24
MD-2 I46	MD-2 I32	MD-2 I32	MD-2 I32
MD-2 V48	MD-2 I44	MD-2 I44	MD-2 I44
MD-2 L61	MD-2 I46	MD-2 I46	MD-2 I46
MD-2 F76	MD-2 V48	MD-2 V48	MD-2 V48
MD-2 R90	MD-2 I52	MD-2 I52	MD-2 I52
MD-2 E92	MD-2 L54	MD-2 L54	MD-2 L54
MD-2 I94	MD-2 L61	MD-2 L61	MD-2 L61
MD-2 Y102	MD-2 I63	MD-2 I63	MD-2 I63
MD-2 I117	MD-2 Y65	MD-2 Y65	MD-2 Y65
MD-2 F119	MD-2 L71	MD-2 L71	MD-2 L71
MD-2 S120	MD-2 L74	MD-2 L74	MD-2 L74
MD-2 F121	MD-2 F76	MD-2 F76	MD-2 F76
MD-2 K122	MD-2 L78	MD-2 L78	MD-2 L78
MD-2 I124	MD-2 I80	MD-2 I80	MD-2 I80
MD-2 F126	MD-2 L87	MD-2 L87	MD-2 L87
	MD-2 R90	MD-2 R90	MD-2 R90
	MD-2 E92	MD-2 E92	MD-2 E92
	MD-2 I94	MD-2 I94	MD-2 V93
	MD-2 Y102	MD-2 Y102	MD-2 I94
	MD-2 F104	MD-2 F104	MD-2 Y102
	MD-2 V113	MD-2 V113	MD-2 F104
	MD-2 T115	MD-2 T115	MD-2 V113
	MD-2 I117	MD-2 I117	MD-2 T115
	MD-2 S118	MD-2 S118	MD-2 I117
	MD-2 F119	MD-2 F119	MD-2 S118
	MD-2 S120	MD-2 S120	MD-2 F119
	MD-2 F121	MD-2 F121	MD-2 S120
	MD-2 K122	MD-2 K122	MD-2 F121
	MD-2 G123	MD-2 G123	MD-2 K122
	MD-2 I124	MD-2 I124	MD-2 G123
	MD-2 F126	MD-2 F126	MD-2 I124
	MD-2 C133	MD-2 C133	MD-2 F126
	MD-2 V135	MD-2 V135	MD-2 C133
	MD-2 F147	MD-2 F147	MD-2 V135
	MD-2 L149	MD-2 L149	MD-2 L146
	MD-2 F151	MD-2 F151	MD-2 F147
	MD-2 I153	MD-2 I153	MD-2 L149
			MD-2 F151
			MD-2 I153

Table S4: Physicochemical characteristics of dispersions prepared in HEPES buffer (5 mM, pH 7.4). Results denote mean values ± SD (n=3).

**Formulation**	***z*-average (nm)**	**PDI**	**Zeta potential (mV)**	**Entrapment efficiency (%)**	**siRNA loading (µg siRNA/mg NPs)**
L_5 SNALPs[a]	79.4 ± 1.2***	0.086 ± 0.020	-15.4 ± 1.1***	95.3 ± 4.2***	128.7 ± 5.6***
L_{mix} SNALPs[a]	85.9 ± 5.5***	0.074 ± 0.038	-10.0 ± 1.5***	81.9 ± 2.1*	110.6 ± 2.8***
L_4 LPNs[b]	204.3 ± 14.1	0.135 ± 0.024	26.8 ± 8.9	61.4 ± 2.8**	4.6 ± 0.2**
L_5 LPNs[b]	212.5 ± 11.5	0.121 ± 0.027	23.2 ± 5.1	72.5 ± 3.8	5.4 ± 0.3
L_6 LPNs[b, d]	286.7 ± 23.0***	0.375 ± 0.027***	3.6 ± 2.9**	75.5 ± 3.6	5.7 ± 0.3
L_{mix} LPNs[b]	201.0 ± 8.5	0.129 ± 0.019	32.8 ± 5.7	52.5 ± 2.9***	3.9 ± 0.2***
DOTAP LPNs[c]	192.7 ± 16.4	0.110 ± 0.013	26.5 ± 4.1	28.3 ± 2.3***	2.1 ± 0.2***

[a] Prepared at an siRNA:lipid weight ratio of 1:7.5. [b] Prepared at 15% (w/w) lipidoid content at an siRNA:lipidoid weight ratio of 1:20; [c] Prepared at 10% (w/w) lipid content an siRNA:lipid weight ratio of 1:10; [d] A bimodal particle size distribution was noted. Statistically significant differences from L_5-modified LPNs are indicated: *$p < 0.05$, **$p < 0.01$ and ***$p < 0.001$.

Table S5: **siRNA sequences and modification patterns**. Lower case letters represent deoxyribonucleotides, underlined capital letters represent 2´-O-methylribonucleotides, and p denotes a phosphate residue.

**Name**	**Target GenBank**	**Sense sequence**	**Antisense sequence**
siRNA-EGFP	JQ064510.1	5'-pACCCUGAAGUUCAUCUGCACCACcg-3'	5'-CGGUGGUGCAGAUGAACUUCAGGUCA-3'
Neg control		5'-AUCGUACGUACCGUCGUAUtt-3'	5'-AUACGACGGUACGUACGAUtt-3'
